# Acute Kidney Injury Secondary to Rhabdomyolysis: A Case of Child Physical Abuse

**DOI:** 10.7759/cureus.33719

**Published:** 2023-01-12

**Authors:** Abdulaziz S Alrafiaah, Manal Alsaiari, Khamisa Almokali, Abdullah T Al Qahtani

**Affiliations:** 1 Department of Pediatrics, College of Medicine, Majmaah University, Majmaah, SAU; 2 Department of Pediatrics, King Abdullah Specialized Children's Hospital, Riyadh, SAU

**Keywords:** childhood maltreatment, non-accidental trauma, acute kidney injury, child abuse, rhabdomyolysis

## Abstract

Child abuse is a challenging problem that any healthcare worker might encounter. It can lead to multiple physical and psychological effects on the child. We report a case of an eight-year-old boy who presented to the emergency department with history of decreased level of consciousness and change in urine color. On examination, he was found to be jaundiced, pale, and hypertensive (160/90 mmHg) with multiple skin abrasions all over the body, suggestive of physical abuse. Laboratory investigations were consistent with acute kidney injury and significant muscle damage. The patient was admitted to intensive care unit (ICU) as a case of acute renal failure secondary to rhabdomyolysis, and subsequently required temporary hemodialysis during his stay in the ICU. The child protective team was involved in the case throughout his hospital admission. Rhabdomyolysis with acute kidney injury secondary to child abuse is an unusual presentation in children, and reporting such cases may lead to early diagnosis and initiation of prompt interventions.

## Introduction

Despite the remarkable progress which medicine reached nowadays, recognition of abused children is considered a major challenge for healthcare providers all over the world [1]. The incidence of child abuse cases does not reflect the actual extent of this problem, as most of the cases go unreported [2]. Bruises, bites, skeletal fractures, and burns are common examples of injuries observed in children who are victims of physical abuse [3]. One of the complications of physical abuse is rhabdomyolysis, which is most commonly caused in children by viral myositis and trauma [4-5]. Few case reports in the literature reported an association between child physical abuse and the development of acute kidney injury due to rhabdomyolysis [6-8]. To our knowledge, this is the second case report in Saudi Arabia with similar findings [6].

## Case presentation

An eight-year-old boy presented to the emergency department of a tertiary hospital in Riyadh with a history of decreased level of consciousness, change in urine color, and recurrent vomiting for one day. Upon presentation to the hospital, physical examination revealed a lethargic but arousable child, who was ill-looking, pale, dehydrated, and jaundiced. His vital signs showed severe hypertension (160/90 mmHg) and heart rate (HR) 140 tachycardia, and growth parameters were below the third percentile. No edema was noted. There were multiple abrasions all over his body, and a human adult bite mark was detected on the left upper back. No hepatosplenomegaly was noted. Other systemic examinations were unremarkable. Perineal examination was normal with no abrasion or other signs suggesting sexual abuse.

While interviewing the child, it was determined that he is originally from another city, and comes from a separated family. He was forced into manual labor, where his employer used to beat him on a daily basis because he could not work properly. He was beaten by sticks and pinched by pliers on all of his body. Three days before he presented to the hospital, he was restrained by his employer and was beaten aggressively, and no food or drink was provided to him. He was finally brought by his cousin to the hospital when he developed emesis and change in mentation.

The initial laboratory results revealed a creatine kinase of more than 42670 U/L, blood urea nitrogen (BUN) 43.4 mmol/L, and creatinine 430 pmol/L. Coagulation profile was normal. The remainder the patient’s laboratory results are summarized in Tables 1-2.

**Table 1:** Urine analysis. UA pH, pH of urine; UA glucose, glucose in urine; UA RBC, red blood cells in urine; Ubg, urobilinogen

Gravity	1.006
UA pH	7
Leu	25
Nit	Negative
Pro	20
UA Glucose	100
Ket	Negative
Ubg	Normal
UA RBC	122

**Table 2:** Initial laboratory results. ALT, alanine aminotransferase; AST, aspartate aminotransferase; ALP, alkaline phosphatase; BNP, brain natriuretic peptide

Test	Result
Hemoglobin	95 g/L
White blood count	20.50 × 10^9^/L
Platelets	331/L
Sodium	129 mmol/L
Potassium	7.6 mmol/L
Chloride	102 mmol/L
Calcium	1.8 mmol/L
Phosphorus	3.39 mmol/L
Magnesium	1.37 mmol/L
BUN	43.4 mmol/L
Creatinine	430 pmol/L
ALT	897 U/L
AST	796 U/L
ALP	155 U/L
Creatine kinase	> 42670 U/L
Total serum bilirubin	22.0 pmol/L
Total protein	55 g/L
Albumin	35 g/L
Uric acid	1216 pmol/L
BNP	242 pmol/L
Troponin	564 pg/mL

Due to his suspected traumatic injuries, multiple imaging studies were obtained. A brain CT scan did not show any acute brain insult. A chest CT showed multiple non-displaced posterior rib fractures with minimal bilateral pleural effusions and bilateral lungs small contusions. An abdominal ultrasound was unremarkable. The patient was then admitted to the intensive care unit (ICU) as case of acute kidney injury secondary to rhabdomyolysis. Severe hyperkalemia was managed medically without the need for the dialysis.

During his stay in the ICU, renal function deteriorated despite optimized conservative management and hemodialysis started due to metabolic complications. After hemodialysis, he recovered from the acute kidney injury and his renal function normalized. Dialysis was stopped after 4 days. Repeated liver profile, muscle enzymes, and blood counts all returned to normal limits.

After the child improved, he was transferred to the general ward with continued improvement. The discharge of the patient was arranged with the hospital’s child protective team-who were involved in the case throughout the patient’s hospital stay-to discharge the patient with a reliable caretaker after reviewing his entire case with relevant governmental authorities.

## Discussion

Rhabdomyolysis is a pathological condition where skeletal muscles are damaged and internal contents of the cells are released into the systemic circulation [9]. Myoglobin, creatine phosphokinase (CK), and lactate dehydrogenase are the main components which indicate significant muscle damage [4]. Myoglobin can induce renal dysfunction by three methods as reported in the literature: i) it is a potent renal vasoconstrictor, ii) leads to the formation of intratubular casts, iii) and has a direct toxic effect on the proximal tubules leading to acute tubular necrosis [10-11]. 

Rhabdomyolysis can result from traumatic muscle injury which occurs secondary to child physical abuse. Such cases require a high index of suspicion, in addition to the relevant information that are obtained from history, physical examination, and investigations to reach the diagnosis. In pediatric patients a study was conducted in Korea which indicated that the most common cause of rhabdomyolysis in children was infection, followed by trauma or surgery and prolonged convulsions [12]. 

Rhabdomyolysis presents a continuum of problems leading to renal injury. In cases of traumatic rhabdomyolysis, initial intravascular volume depletion (pre-renal syndrome) will potentiate the nephrotoxic effects of heme-pigments (Hgb, myoglobin) on the proximal tubule. Rhabdomyolysis resulting in acute kidney injury was supported by the change in the child’s urine color, in addition to the results of laboratory investigations which indicted a significant muscle damage with evidence of acute kidney injury. The patient's high liver enzymes can be attributed not only to the liver injury also it can occur with rhabdomyolysis itself. One study found that increased aspartate aminotransferase level and reduced calcium levels were associated with development of acute kidney injury in patient with rhabdomyolysis [12]. Those factors also were found in our case.

The acute kidney injury was severe enough that the patient required hemodialysis as he did not improve with conservative measures. Also, deprivation of water and food for three days before the patient was brought to the hospital itself may cause kidney injury. This might be an additional reason why acute kidney injury in this case was so severe as to require renal replacement therapy. Unfortunately, children who develop acute kidney injury requiring dialysis put them at a high risk of developing future renal related complications, such as end stage renal disease [13]. 

Furthermore, the extent of damage in our patient was not only confined to the kidneys. Other investigations showed diffuse axonal injury, multiple ribs factures, liver injury, and bilateral lung contusions which were all managed conservatively. Such findings are common to be observed in cases of child physical abuse [14]. 

Child abuse is a major problem which affects children’s lives around the world. It has long-term physical and psychological consequences on the affected child, and it can be the cause of death for those children. In Saudi Arabia, several cases of child abuse were reported, however, only one study reported a similar presentation, where the child had acute kidney injury following rhabdomyolysis which occurred as result of child physical abuse [6]. Additionally, a case was reported in 2021 about an infant who had traumatic rhabdomyolysis following severe physical abuse [15]. One study from Saudi Arabia, evaluated child abuse incidences and its associated risk factors during COVID-19 pandemic, and the results showed significant changes as compared to the period before pandemic [16].

Victims of child abuse can have multiple injuries involving various body systems, which mandates a careful and thorough assessment [17]. Acute kidney injury and its related complications require prompt treatment in order to decrease morbidity and mortality.

## Conclusions

Rhabdomyolysis complicated by acute kidney injury following child physical abuse is a life threatening and unusual presentation among children. Early detection and initiation of appropriate management are imperative. A thorough clinical assessment and a high index of suspicion are keys to identifying such cases.
